# Firearm Availability and Storage Practices Among Military Personnel Who Have Thought About Suicide

**DOI:** 10.1001/jamanetworkopen.2019.9160

**Published:** 2019-08-16

**Authors:** Craig J. Bryan, AnnaBelle O. Bryan, Michael D. Anestis, Lauren R. Khazem, Julia A. Harris, Alexis M. May, Cynthia Thomsen

**Affiliations:** 1 The University of Utah, Salt Lake City; 2 National Center for Veterans Studies, Salt Lake City, Utah; 3 University of Southern Mississippi, Hattiesburg; 4 Wesleyan University, Middletown, Connecticut; 5 Naval Health Research Center, San Diego, California

## Abstract

This cross-sectional study examines firearm ownership and storage practices among US military personnel who have experienced thoughts of suicide.

## Introduction

More than 60% of US military suicides occur at home and involve a firearm.^1^ Nearly all military firearm suicides (95%) involve a personally owned firearm.^1^ Nonmilitary data indicate that the risk of suicide is 6 times higher in households with a firearm, although this risk may be reduced if the firearms are kept unloaded and/or locked.^2^ Because attempts using firearms have very high fatality rates,^3^ safe firearm storage practices could be an important component of comprehensive suicide prevention in the military. This study examined associations of firearm ownership and storage practices with suicidal thoughts and behaviors among military personnel.

## Methods

In a cross-sectional study, we examined firearm storage practices among 1652 active-duty military personnel enrolled in the Primary Care Screening Methods (PRISM) study, conducted in 6 military primary care clinics across the United States between July 13, 2015, and August 22, 2018. Service members who were eligible for military medical services, aged 18 years or older, and able to complete informed consent procedures completed self-report measures during routine clinic visits. The study was approved by the Naval Health Research Center’s institutional review board, and participants provided written informed consent. This study followed the Strengthening the Reporting of Observational Studies in Epidemiology (STROBE) reporting guideline.

Firearm ownership and storage practices were assessed using Behavioral Risk Factor Surveillance System items.^4^ All participants were asked, “Are any firearms now kept in or around your home?” Those responding affirmatively were subsequently asked, “Are any of these firearms now loaded?” and “Are any of these firearms now unlocked?” Safe storage was defined as having firearms locked up and unloaded. Lifetime history of suicide ideation and attempts was assessed using items from the Self-injurious Thoughts and Behaviors Interview^5^: “Have you ever had thoughts of killing yourself?” and “Have you ever made an actual attempt to kill yourself in which you had at least some intent to die?” Thoughts of death or self-harm during the preceding 2 weeks were assessed using item 9 of the Patient Health Questionnaire 9.^6^

To test associations among variables, SPSS statistical software version 25 (IBM) was used to calculate adjusted odds ratios with 95% confidence intervals. Statistical significance was set at *P* < .05 using 2-sided tests.

## Results

Of 1652 participants (1071 [64.8%] male; mean [SD] age, 33.6 [15.7] years), 590 participants (35.7%) reported a firearm in or around their home, 141 (8.6%) selected “refuse to answer” or skipped the item, and 11 (0.1%) selected “I don’t know.” Among participants with a firearm in or around the home, 124 (21.0%) indicated their firearms were loaded and unlocked, 188 (32.2%) indicated their firearms were safely stored (ie, unloaded and locked up), 150 (25.3%) indicated their firearms were not safely stored (ie, 60 [10.2%] unloaded but not locked up and 90 [15.3%] locked up but loaded), and 126 [21.3%] refused to answer or skipped the items. Factors associated with firearm access and safe storage are summarized in Table 1 and Table 2. Participants with recent thoughts of death or self-harm were significantly less likely to have a firearm at home (odds ratio, 0.61; 95% CI, 0.40-0.95; *P* = .03). However, among those with a firearm at home, safe storage was less common among participants endorsing a lifetime history of suicide ideation (odds ratio, 0.47; 95% CI, 0.29-0.78; *P* = .003) or recent thoughts about death or self-harm (odds ratio, 0.26; 95% CI, 0.09-0.79; *P* = .02).

**Table 1.** Demographic Differences Between Military Personnel With and Without a Firearm Located in or Around the Home^a^

Characteristic	Firearm at Home, No. (%)		Adjusted Odds Ratio (95% CI)	*P* Value
	No (n = 910)	Yes (n = 590)		
Age, mean (SD), y	32.0 (15.1)	35.1 (16.1)	1.01 (1.00-1.02)	.10
Sex				
Male	549 (60.9)	422 (71.8)	0.78 (0.61-1.01)	.06
Female	352 (39.1)	166 (28.2)	1 [Reference]	NA
Race/ethnicity				
White				
No	387 (43.0)	127 (21.5)	1 [Reference]	NA
Yes	514 (57.0)	461 (78.4)	1.87 (1.27-2.75)	.001
Black				
No	658 (73.0)	520 (88.4)	1 [Reference]	NA
Yes	243 (27.0)	68 (11.6)	0.52 (0.34-0.79)	.002
Asian				
No	854 (94.8)	568 (96.6)	1 [Reference]	NA
Yes	47 (5.2)	20 (3.4)	0.72 (0.41-1.27)	.26
Native American				
No	849 (94.2)	558 (94.9)	1 [Reference]	NA
Yes	52 (5.8)	30 (5.1)	0.98 (0.62-1.58)	.95
Pacific Islander				
No	886 (98.3)	582 (99.0)	1 [Reference]	NA
Yes	15 (1.7)	6 (1.0)	0.82 (0.31-2.22)	.70
Hispanic				
No	719 (79.8)	487 (82.8)	1 [Reference]	NA
Yes	182 (20.2)	101 (17.2)	0.91 (0.62-1.33)	.62
Other				
No	772 (85.7)	535 (91.0)	1 [Reference]	NA
Yes	129 (14.3)	53 (9.0)	0.81 (0.49-1.33)	.40
Military branch				
Air Force	62 (6.9)	34 (5.8)	1 [Reference]	NA
Army	154 (17.1)	90 (15.3)	1.29 (0.77-2.16)	.34
Marines	149 (16.5)	104 (17.7)	1.84 (1.09-3.10)	.02
Navy	536 (59.5)	359 (61.1)	1.45 (0.91-2.29)	.12
Ever deployed				
No	396 (55.2)	178 (69.0)	1 [Reference]	NA
Yes	505 (44.8)	410 (31.0)	1.76 (1.33-2.32)	<.001
Suicide risk indicator				
Lifetime suicide ideation				
No	652 (73.1)	412 (70.5)	1 [Reference]	NA
Yes	240 (26.9)	172 (29.5)	1.21 (0.91-1.61)	.20
Lifetime suicide attempt				
No	811 (91.6)	541 (93.4)	1 [Reference]	NA
Yes	74 (8.4)	38 (6.6)	0.81 (0.49-1.32)	.39
Recent thoughts of death or self-harm				
No	814 (90.3)	547 (93.0)	1 [Reference]	NA
Yes	87 (9.7)	41 (7.0)	0.61 (0.40-0.95)	.03

Abbreviation: NA, not applicable.

^a^ Based on all variables entered into the model simultaneously to estimate the presence of a firearm at home.

**Table 2.** Demographic and Clinical Factors Associated With Firearm Storage Practices^a^

Characteristic	Firearm Storage Method, No. (%)		Adjusted Odds Ratio (95% CI)	*P* Value
	Unsafe (n = 274)	Safe (n = 187)		
Age, mean (SD), y	33.3 (15.2)	37.3 (17.1)	1.01 (1.00-1.03)	.06
Sex				
Male	199 (72.6)	136 (72.7)	1.28 (0.80-2.05)	.30
Female	75 (27.4)	51 (27.3)	1 [Reference]	NA
Race/ethnicity				
White				
No	55 (20.1)	46 (24.6)	1 [Reference]	NA
Yes	219 (79.9)	141 (75.4)	0.82 (0.38-1.77)	.61
Black				
No	241 (88.0)	165 (88.2)	1 [Reference]	NA
Yes	33 (12.0)	22 (11.8)	0.91 (0.37-2.21)	.83
Asian				
No	267 (97.4)	178 (95.2)	1 [Reference]	NA
Yes	7 (2.6)	9 (4.8)	1.76 (0.54-5.70)	.35
Native American				
No	262 (95.6)	180 (96.3)	1 [Reference]	NA
Yes	12 (4.4)	7 (3.7)	1.09 (0.39-2.99)	.87
Pacific Islander				
No	273 (99.6)	184 (98.4)	1 [Reference]	NA
Yes	1 (0.4)	3 (1.6)	4.90 (0.61-39.15)	.13
Hispanic				
No	224 (83.6)	153 (83.4)	1 [Reference]	NA
Yes	45 (16.4)	31 (16.6)	1.47 (0.76-2.85)	.25
Other				
No	251 (91.6)	165 (88.2)	1 [Reference]	NA
Yes	23 (8.4)	22 (11.8)	1.55 (0.62-3.86)	.35
Military branch				
Air Force	10 (3.6)	16 (8.6)	1 [Reference]	NA
Army	40 (14.6)	33 (17.6)	0.50 (0.19-1.31)	.16
Marines	48 (17.5)	33 (17.6)	0.57 (0.21-1.53)	.27
Navy	176 (64.2)	105 (56.1)	0.37 (0.15-0.88)	.03
Ever deployed				
No	86 (31.4)	51 (27.3)	1 [Reference]	NA
Yes	188 (68.6)	136 (72.7)	1.29 (0.79-2.11)	.31
Suicide risk indicator				
Lifetime suicide ideation				
No	177 (65.1)	147 (78.6)	1 [Reference]	NA
Yes	95 (34.9)	40 (21.4)	0.47 (0.29-0.78)	.003
Lifetime suicide attempt				
No	251 (93.3)	177 (95.2)	1 [Reference]	NA
Yes	18 (6.7)	9 (4.8)	2.01 (0.75-5.39)	.17
Recent thoughts of death or self-harm				
No	249 (90.9)	183 (97.9)	1 [Reference]	NA
Yes	25 (9.1)	4 (2.1)	0.26 (0.09-0.79)	.02

Abbreviation: NA, not applicable.

^a^ Based on all variables entered into the model simultaneously to estimate safe firearm storage.

## Discussion

In this cross-sectional study of a sample of active-duty military personnel, one-third reported a firearm in or around the home. Of this subgroup, one-third reported storing the firearm safely (ie, unloaded and locked up). Although military personnel with recent thoughts about death or self-harm were less likely to have a firearm at home, suicidal personnel who did have a firearm at home were much less likely than nonsuicidal service members to use safe storage. This highlights the importance of emphasizing safe storage of personally owned firearms, including temporary removal of access to firearms for high-risk personnel. Limitations of this study include self-report methods, cross-sectional design, and unknown response rate. Further research focused on firearm availability and storage practices among military personnel is warranted.
